# Supplementary material for: Radiation-dose effects in correlative X-ray/cryo-electron microscopy of frozen-hydrated biological samples
Source: Acta Crystallogr D Struct Biol. 2026 Feb 26;82(Pt 3):207–15. doi: 10.1107/S2059798326001427 (PMC12954863; doi:10.1107/S2059798326001427)
Supplement: Supplementary file 1 [file d-82-00207-sup1.pdf]

Acta Cryst D STRUCTURAL BIOLOGY

Volume 82 (2026)

Supporting information for article:

Radiation-dose effects in correlative X-ray/cryo-electron microscopy of frozen-hydrated biological samples

Thorsten B. Blum, Vincent Olieric, Ana Diaz, Takashi Ishikawa and Volodymyr M. Korkhov

A

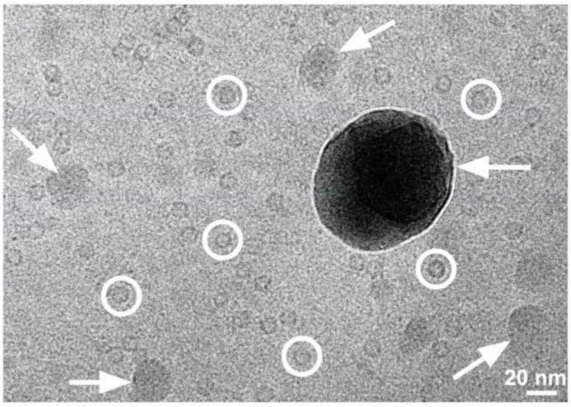

B

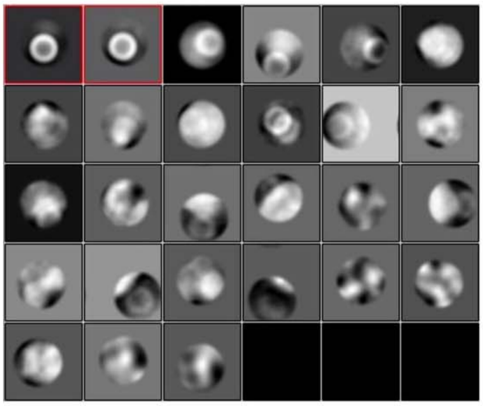

C	0 MGy	1 MGy	100 MGy
Recorded	760 movies	405 movies	565 movies
Autopicking	60,105 particles	48,194 particles	80,664 partcìles
4 × 2D classification	33,386 particles	8,548 particles	13,728 particles
1st 3D classification	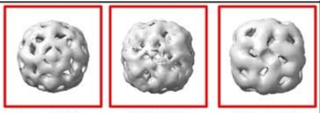 24.3% 36.5% 39.2%<br>33,386 particles	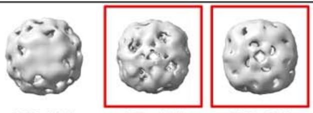 22.0% 43.1% 34.9%<br>6,674 particles	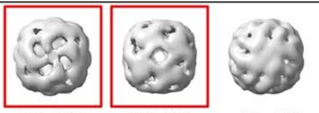 32.5% 54.1% 13.4%<br>9,547 particles
2nd 3D classification	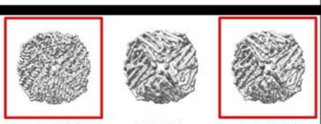 46.5% 9.5% 44.0%<br>30,265 particles	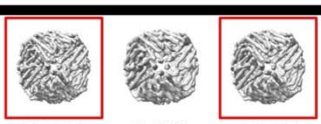 50.8% 2.8% 46.4%<br>6,488 particles	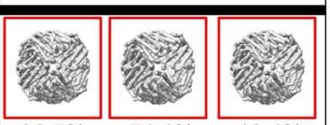 32.5% 54.1% 13.4%<br>9,547 particles
3D refinement	3.8 Å<br>30,265 particles	4.3 Å<br>6,488 particles	4. Å<br>9,547 particles
CTF refinement	3.4 Å<br>30,265 particles	3.9 Å<br>6,488 particles	4.3 Å<br>9,547 particles
Polish	3.2 Å<br>30,265 particles	3.8 Å<br>6,488 particles	4.1 Å<br>9,547 particles
3D refinement	3.7 Å<br>6,488 particles	3.8 Å<br>6,488 particles	4.3 Å<br>6,488 particles
Postprocess	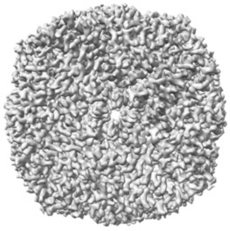 3.17 Å<br>6,488 particles	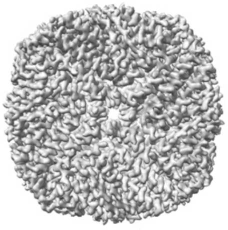 3.56 Å<br>6,488 particles	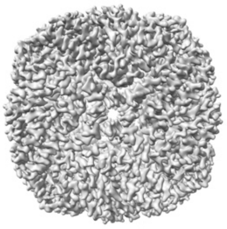 3.88 Å<br>6,488 particles

Figure S1 Processing pipeline of the three apoferritin data sets. (A) Representative micrograph showing apoferritin particles (circles) and regions of variable ice contamination (arrows). (B) Particles were template-picked and subjected to iterative 2D classification. Only classes with clear apoferritin features (red boxes) were retained. (C) Initial 2D and 3D classifications were performed with binned particles, followed by re-extraction at full resolution, final 3D classification, CTF refinement, and polishing. For comparison, each data set was equalized to 6,488 particles before final refinement and post-processing, yielding a resolution of 3.88 Å even after absorbing 100 MGy X-ray dose.

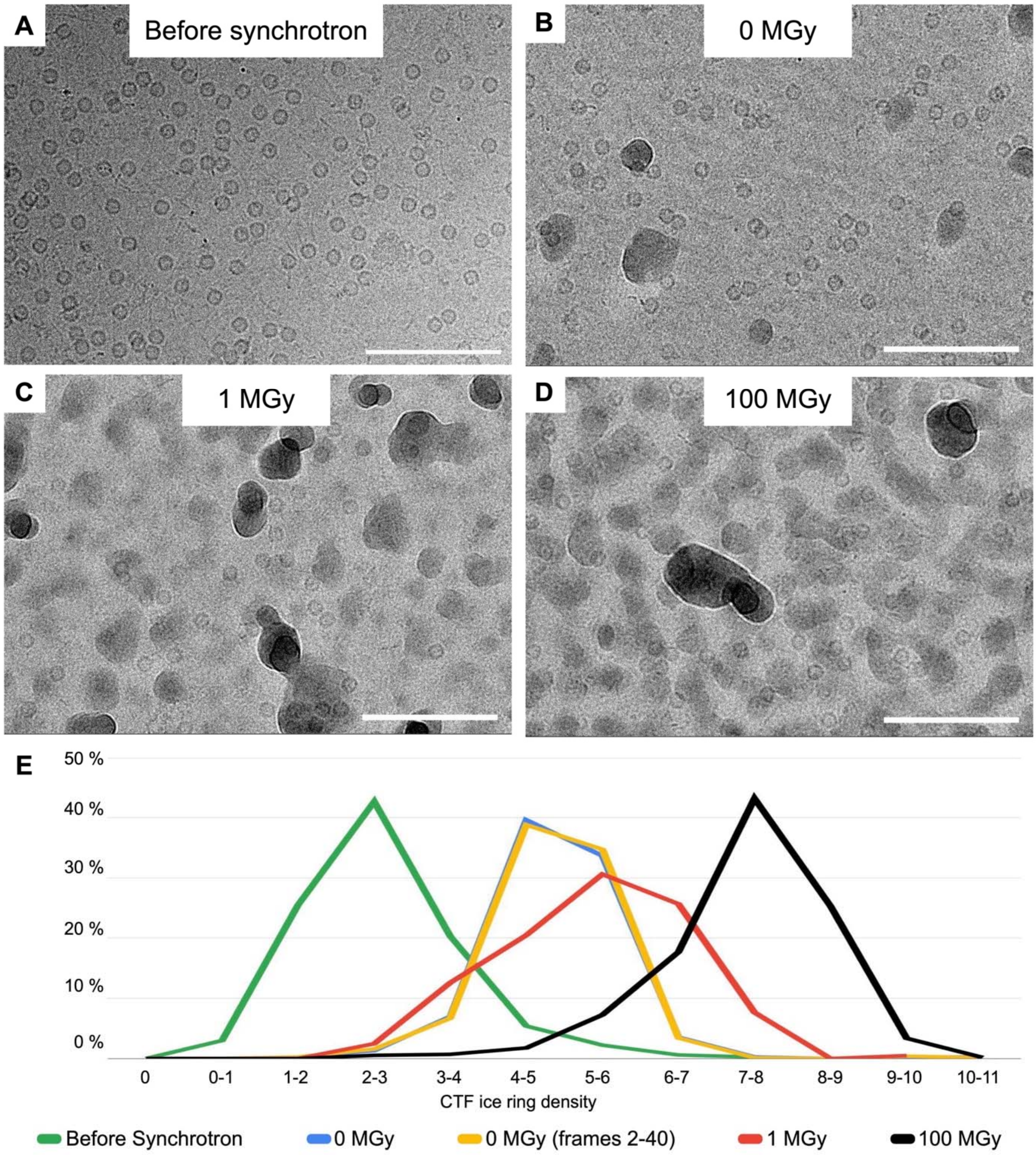

Figure S2 Ice contamination of samples. (A) Prior to synchrotron exposure, the sample displayed minimal crystalline ice. (B) After synchrotron handling, non-exposed regions showed increased ice, while areas that had absorbed X-ray doses of (C) 1 MGy or (D) 100 MGy exhibited even greater contamination (scale bars: 100 nm). (E) Quantification of CTF ice ring density using CTFFIND confirmed these observations, indicating that synchrotron handling promotes ice accumulation, which intensifies under X-ray irradiation.

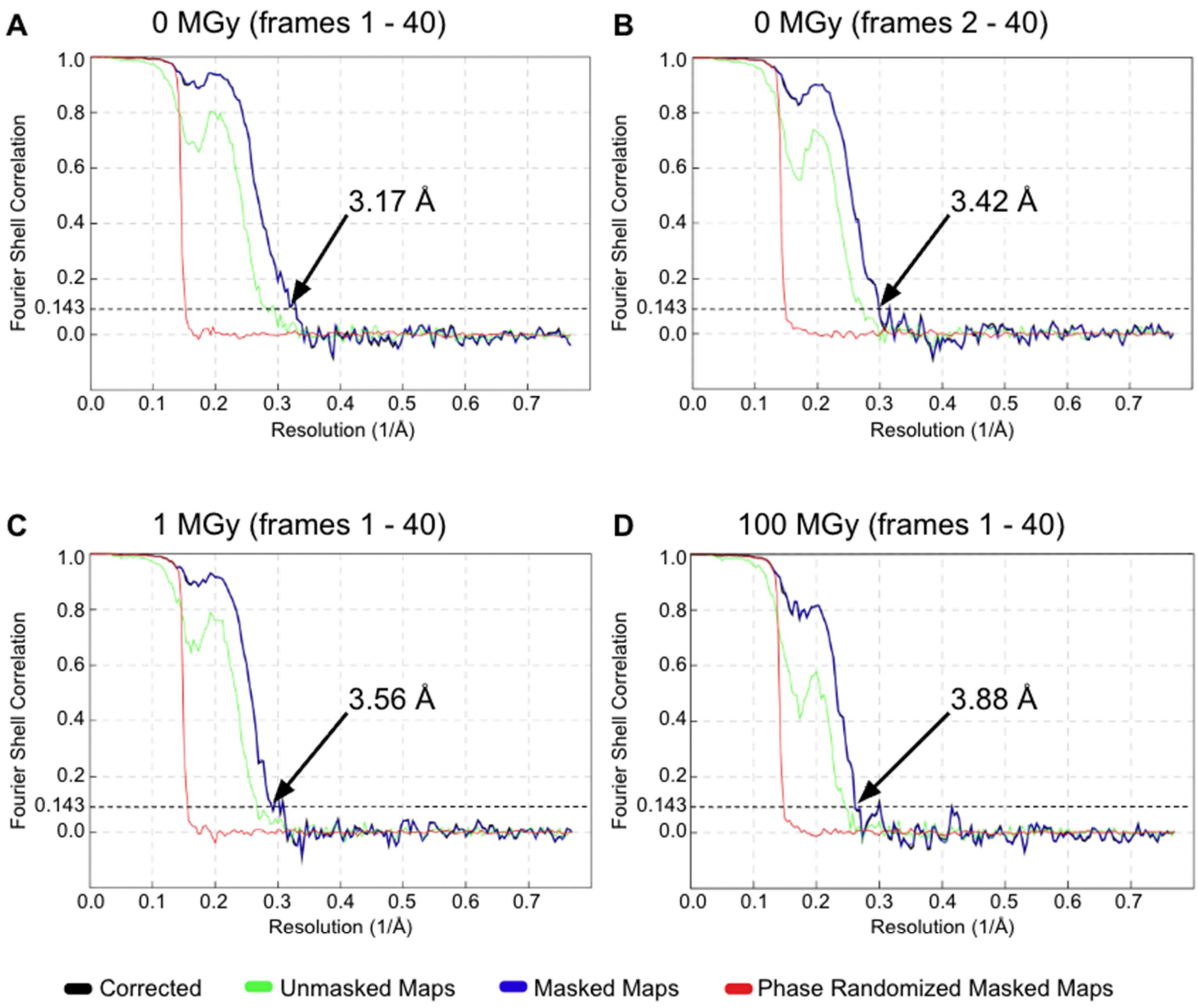

Figure S3 Fourier shell correlations. Fourier shell correlation (FSC) plots of apoferritin from a grid square not exposed to X-rays, using either (A) all frames or (B) excluding the first frame, yielded the highest resolution. In contrast, FSC plots from grid squares exposed to X-ray doses of (C) 1 MGy or (D) 100 MGy showed reduced resolution.
